# Peripheral blood CD34+ peak during mobilisation as an additional parameter to assess PBSC inoculum adequacy autologous HSCT

**DOI:** 10.3389/fimmu.2026.1935217

**Published:** 2026-10-16

**Authors:** Giuseppe Milone, Emanuele Martorana, Maria Grazia Camuglia, Salvatore Leotta, Andrea Spadaro, Giulia Giuffrida, Gaetano Moschetti, Federica Galbo, Paola Scirè, Giulio Antonio Milone, Giordana Brunetti, Paolo Castorina

**Affiliations:** 1 Istituto Oncologico del Mediterraneo, Hematopoietic Stem Cell Transplantation (HSCT) Unit-Viagrande, Viagrande, Italy; 2 Programma di Trapianto Emopoietico - Hematopoietic Stem Cell Transplantation (HSCT) Unit-Policlinico di Catania, Catania, Italy; 3 Department of Experimental Medicine (DIMES), University of Genoa, Genova, Italy; 4 Istituto Nazionale Fisica Nucleare, Catania, Italy; 5 Institute of Particle and Nuclear Physics, Charles University, Prague, Czechia

**Keywords:** CD34 cells +, engraftment, HSC transplantation, mobilisation, PBSC apheresis

## Abstract

**Background:**

After autologous haematopoietic stem cell transplantation (HSCT), the infused CD34+ cell dose is usually considered essential for assessing the adequacy of the peripheral blood progenitor cells (PBSC) inoculum.

**Methods:**

In a cohort of 302 patients who underwent autologous haematopoietic transplantation, we investigated whether the peak peripheral blood CD34+ cell count during mobilisation could predict neutrophil engraftment.

**Results:**

In the overall cohort, the mean time to neutrophil engraftment was 12.2 days, and the mean infused CD34+ cell dose was 5.1 × 10^6 CD34+ cells/kg. In univariate analysis, the CD34+ cell dose (p=0.005), the PB CD34+ peak during mobilisation (p=0.0002), the number of performed apheresis (p=0.03), and the use of G-CSF (p=0.001) were significantly associated with time to neutrophil engraftment. When factors were evaluated in multivariate analysis, the PB CD34+ peak and use of G-CSF remained significant. In a ROC analysis, the PB CD34+ peak predicted delayed engraftment with an AUC of 0.695 (95% CI, 0.599-0.791). Joint stratification by PB CD34+ peak and infused CD34+ dose identified subgroups at high risk of delayed engraftment.

**Conclusion:**

In patients treated by autologous HSCT and receiving an adequate dose of infused CD34+ cells, the PB CD34+ peak during mobilisation is independently associated with post-transplant neutrophil engraftment.

## Introduction

Autologous transplantation of haematopoietic stem cells (HSCs) has been used for several decades in chemosensitive haematological malignancies to rescue haematopoiesis after high-dose chemoradiotherapy. The procedure carries some risks and has a treatment-related mortality ranging from 1 to 3%. The risks of autologous HSC transplantation include, among others, delayed haematopoietic engraftment of the inoculum (1).

In most centres, the minimum infused CD34+ cell dose considered necessary for prompt haematopoietic recovery is 2 × 10^6/kg. This threshold is based on observations that delayed engraftment is more common when the infused CD34+ cell dose is below 2 × 10^6/kg (2–5). In addition, an even higher CD34+ quota offers several advantages, including faster platelet engraftment times (6).

However, the minimum HSC dose required for reliable rescue is not uniformly defined, and different centres use varying practical thresholds. Some authors have reported normal engraftment regardless of the infused CD34+ cell dose (7).

Additional factors beyond the dose of CD34+ cells may contribute to engraftment times, and their study may help reduce the risk of engraftment failure associated with autologous transplantation. In addition to the number of CD34+ cells infused, the quality of CD34+ cells is considered important for rapid and long-term engraftment (8, 9).

Rapid achievement of the target CD34+ cell dose during apheresis has been considered a surrogate marker of HSC quality, and collecting high CD34+ yields in a single apheresis has been associated with a favourable clinical outcome (10–12).

However, the possibility of collecting a high CD34+ quota in a single apheresis is also influenced by factors unrelated to HSC quality or to biological aspects of bone marrow function, such as the accuracy of patient monitoring during mobilisation, the adequacy of venous access, and the apheresis technique used. Indeed, the CD34+ cell peak observed in PB during mobilisation could be a more reliable index of HSC quality. In fact, this index is influenced only by marrow function and the efficacy of the mobilisation process, and not by the other clinical factors listed above.

We therefore hypothesised that the PB CD34+ peak observed during HSC mobilisation could, by reflecting the overall biology of HSC mobilisation, help in predicting post-transplant engraftment times. Here, we present the results of a study examining the correlation between engraftment time and peak CD34+ cell count in the PB in a case series of more than 300 autologous transplants.

## Patients and methods

Study design: We prospectively collected neutrophil engraftment data from a cohort of autologous transplants and correlated time to engraftment with both the maximum PB CD34+ cell peak observed during mobilisation at day of PBSC harvest and the infused CD34+ cell dose per kg. Since the main factor to be studied was the peak CD34+ cell count in the PB reached during mobilisation, only patients receiving an HSC product collected from a single mobilisation were included. Cases in which autologous transplants were performed using an inoculum combining HSC collected in different mobilisations and at different times were excluded, since in this case the CD34+ peaks in the PB would have been more than one.

Patient features: We studied 302 patients who underwent high-dose chemotherapy and autologous HSC transplantation from January 2015 to December 2024 within the Metropolitan HSC Transplantation Program of Catania (Italy). This accounted for 42% of all autologous transplants performed in our HSCT Program (n = 715) in the same time-frame. Patients were treated either in the IOM Clinical Unit in Viagrande or at the Clinical Unit of the Policlinico-University of Catania. All patients had previously received mobilisation and apheresis in the same Transplant Program. Each patient signed a written informed consent to authorise data collection. The characteristics of the patients included in the study are shown in **Table 1**.

**Table 1:** Demographic and clinical data of patients studied.

	All patients N 302	MM N 213	NHL N 57	HD N 32
Age years (mean)	53.2 IQR 14 Range: 14-72	56.7 IQR 12 Range: 36-72	52.2 IQR 13 Range: 20-69	32.3 IQR 16 Range: 14-69
Type of mobilisation schedule	CTX+G-CSF: 220 DHAP+ G-CSF: 45 Other Chemo+ G-CSF: 32 G-CSF alone: 5	CTX+G-CSF: 209 DHAP+G-CSF: 1 Other Chemo+ G-CSF: 3 G-CSF alone: 0	CTX+G-CSF: 6 DHAP: 35 Other Chemo+ G-CSF: 11 G-CSF alone: 5	CTX+G-CSF: 5 DHAP: 9 Other Chemo+ G-CSF: 18 G-CSF alone: 0
High-dose Chemio	BEAM/FEAM: 84 HD-PAM: 218	BEAM/FEAM: 0 HD-PAM: 213	BEAM/FEAM: 55 HD-PAM: 2	BEAM/FEAM: 29 HD-PAM:3
N apheresis (mean; range)	1,1 (1-3)	1,1 (1-3)	1,2 (1-3)	1.1 (1-2)

CTX, cyclophosphamide; DHAP, Cisplatin, High dose ARA-C, Desametasone; G-CSF, granulocyte-colony stimulating factor; BEAM/FEAM, BCNU or Fotomustine, Etoposide, ARA-C, Melphalan.

Mobilisation schedules, CD34+ peak in PB and Apheresis. Mobilisation schedules used were: CTX + G-CSF; DHAP + G-CSF; G-CSF alone; other chemotherapy plus G-CSF. Plerixafor (PLX) was used in 29 patients (9.6%) according to an algorithm already described (13).

CD34+ monitoring was performed daily by flow cytometry, starting 8–9 days after administration of mobilising chemotherapy (CTX 2gr/m2) or on day 14 with more aggressive chemotherapy (DHAP or CTX 4 gr/m2) (13, 14). To identify the PB CD34+ peak, the Count of PB CD34+ cells was repeated 2–3 times in subsequent day in each patient. The PB CD34 peak recorded on the day of the first apheresis was thus indicated as “PB CD34+ cell peak”. CD34+ cell enumeration was performed by flow cytometry using a DxFLEX flow cytometer (Beckman Coulter Life Sciences, Brea, CA, USA) and fluorochrome-conjugated monoclonal antibodies against CD45 and CD34 (CD45-FITC/CD34-PE; Beckman Coulter Life Sciences). Viable cells were identified by exclusion of 7-AAD-positive events, and CD34+ cells were enumerated according to the ISHAGE gating strategy. If the patient needed PLX, the peak existing in the morning, before PLX administration, was considered.

Apheresis was generally initiated at the CD34+ peak in PB, and 1–3 aphereses were performed per mobilisation. Peripheral blood progenitor cells (PBSC) apheresis was performed using a Fresenius AS104 cell separator, treating the patient’s blood volume twice. In the case of Multiple Myeloma (MM), the ideal target was CD34+ > 4 × 10^6/kg.

High-dose chemotherapy and Autologous Transplantation. Cryopreservation of PBSC was performed in all cases in our laboratory using 10% DMSO and a temperature-controlled method. Storage was carried out in liquid nitrogen at -196 °C.

Patients with MM received high-dose melphalan, while patients with lymphoma received the high-dose BEAM or FEAM regimen. Thawing and reinfusion were performed at the bedside in a controlled water bath. The primary endpoint was time to neutrophil engraftment, defined as the first of 3 consecutive days with an absolute neutrophil count (ANC) > 0.5 × 10^9/L.

A portion of patients (194/302) received prophylactic G-CSF therapy after autologous transplantation from day +5 to neutrophil engraftment. All patients admitted to the HSC transplantation clinical unit at IOM received post-transplant G-CSF, whereas all patients admitted to the HSC transplantation unit at Policlinico di Catania did not receive prophylactic G-CSF.

### Statistical analysis of factors important for engraftment

Different groups’ engraftment times were analysed using a nonparametric test (Mann-Whitney U or Kruskal-Wallis) and a survival analysis (univariate or multivariate Cox proportional hazards). For continuous variables, discrete groups were created by grouping patients at the median or using percentiles.

For the Survival technique, the engraftment day was treated as the event time, and engrafted patients were considered uncensored. Patients not engrafted by day +60 were considered censored at day +60. Patients not engrafted at the last observation were considered censored at that time point.

A multivariable Cox proportional hazards analysis was performed to evaluate the independent effect of each factor. Variables with p < 0.10 in the univariable analysis were included in the multivariable model. In this analysis, the groups were evaluated using the Hazard Ratio (HR) relative to a reference group, and factors with an HR greater than 1.0 indicated a faster engraftment rate. In contrast, factors with an HR < 1.0 were associated with longer engraftment time. Engraftment time was also studied using cumulative incidence analysis and compared using the Gray test.

In additional exploratory analyses, we assessed whether the association between PB CD34+ peak and neutrophil engraftment differed according to post-transplant G-CSF use. To this end, for each G-CSF group, we fitted a linear model with log10-transformed PB CD34+ peak as the dependent variable and engraftment time as the independent variable.

Statistical evaluation was performed using Stat-View 5.0 (CA) and R version 4.1.2 (2021). ROC analysis was performed using a dedicated R package.

## Results

### Patient’s characteristics and engraftment data

A total of 302 patients were mobilised and underwent apheresis harvests; all received an autologous HSCT using only the inoculum obtained from a single mobilisation.

The mean number of infused CD34+ cells was 5.1 x 10^6/kg (range 1.4-26.5), and the median was 4.2 × 10^6/kg. Neutrophil engraftment was observed in all patients except 4, who died of severe complications before engraftment (sepsis); data for these patients were censored at the time of last observation. The mean time to neutrophil engraftment was 11.5 (range 7-24) days in G-CSF prophylactically treated patients and 13.2 days (range 8-48) in patients not treated prophylactically with G-CSF (Mann-Whitney test p= 0.05).

### Association of infused CD34+ cell dose with engraftment

When patients were divided into 3 groups according to the amount of CD34+ cells infused (first group < 3 x 10^6/kg; second group 3.1 to 6.0 x 10^6/kg; third group > 6.1 x 10^6/kg), the three groups had significantly different times to neutrophil engraftment (Kruskal-Wallis test, p=0.0009). The mean time was 12.8 days in the first group (n = 68), 12.4 days in the second group (n = 160), and 11.4 days in the third group (n = 74) (**Figures 1A**, **2A**).

**Figure 1:**
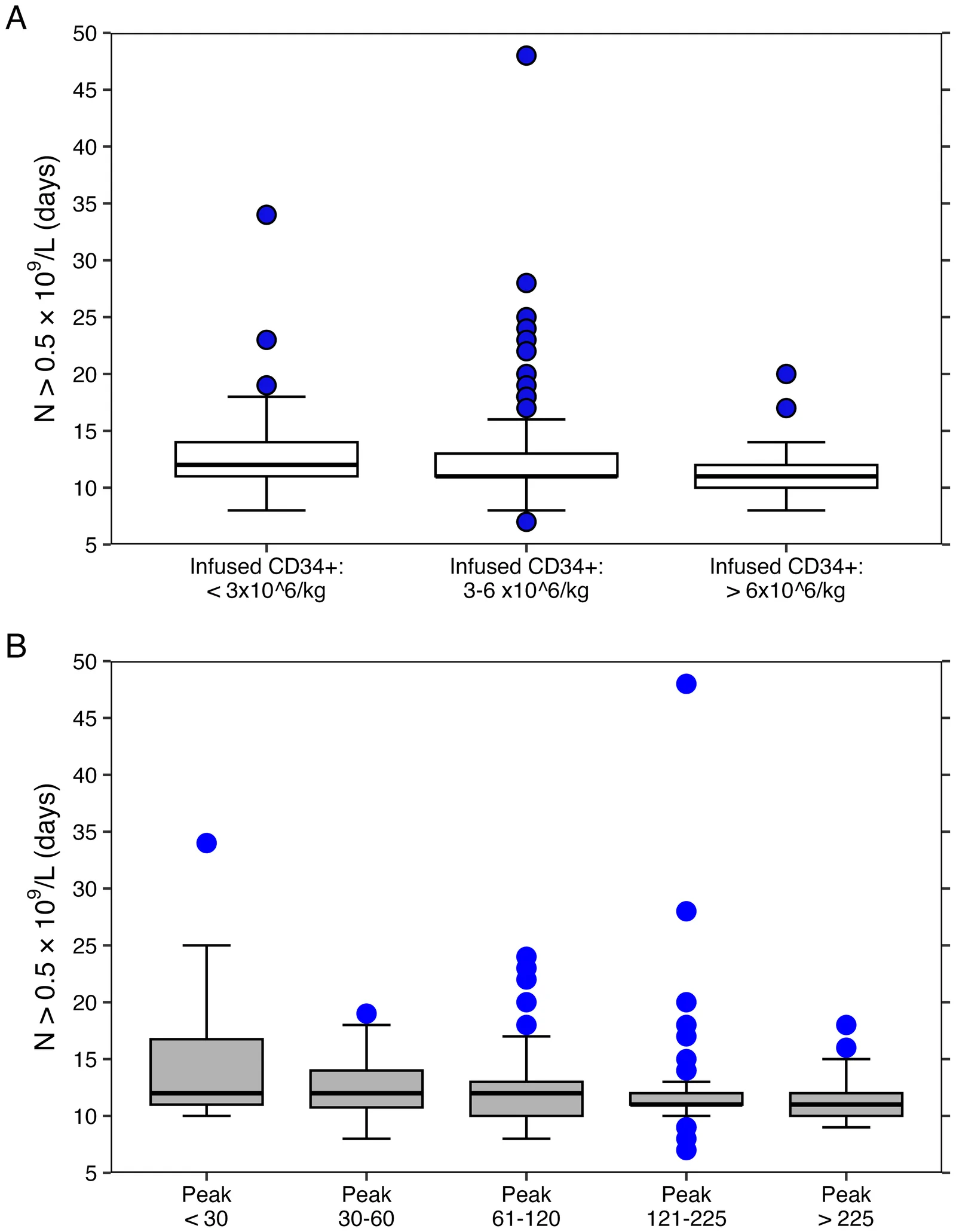
Neutrophil engraftment times in groups defined by the amount of infused CD34+ cells and by the maximum peak of CD34+ cells in PB during CD 34+ mobilisation. Panel **(A)**: Neutrophil engraftment times in groups defined by the amount of infused CD34+ cells. The mean time was 12.8 days in the first group, 12.4 days in the second group, and 11.4 days in the third group. Differences in engraftment time were statistically significant (Kruskal-Wallis test, p=0.0009). Panel **(B)**: Neutrophil engraftment times in groups defined by the maximum peak of CD34+ cells in PB during CD 34+ mobilisation (cells/µL). Neutrophil engraftment in “group A” was 14,6 days; in “group B” it was 12.4 days; in “group C” it was 12.2 days; in “group D” it was 12,7 days, and in “group E” 11,0 days (Kruskal-Wallis test, p=0.001).

**Figure 2:**
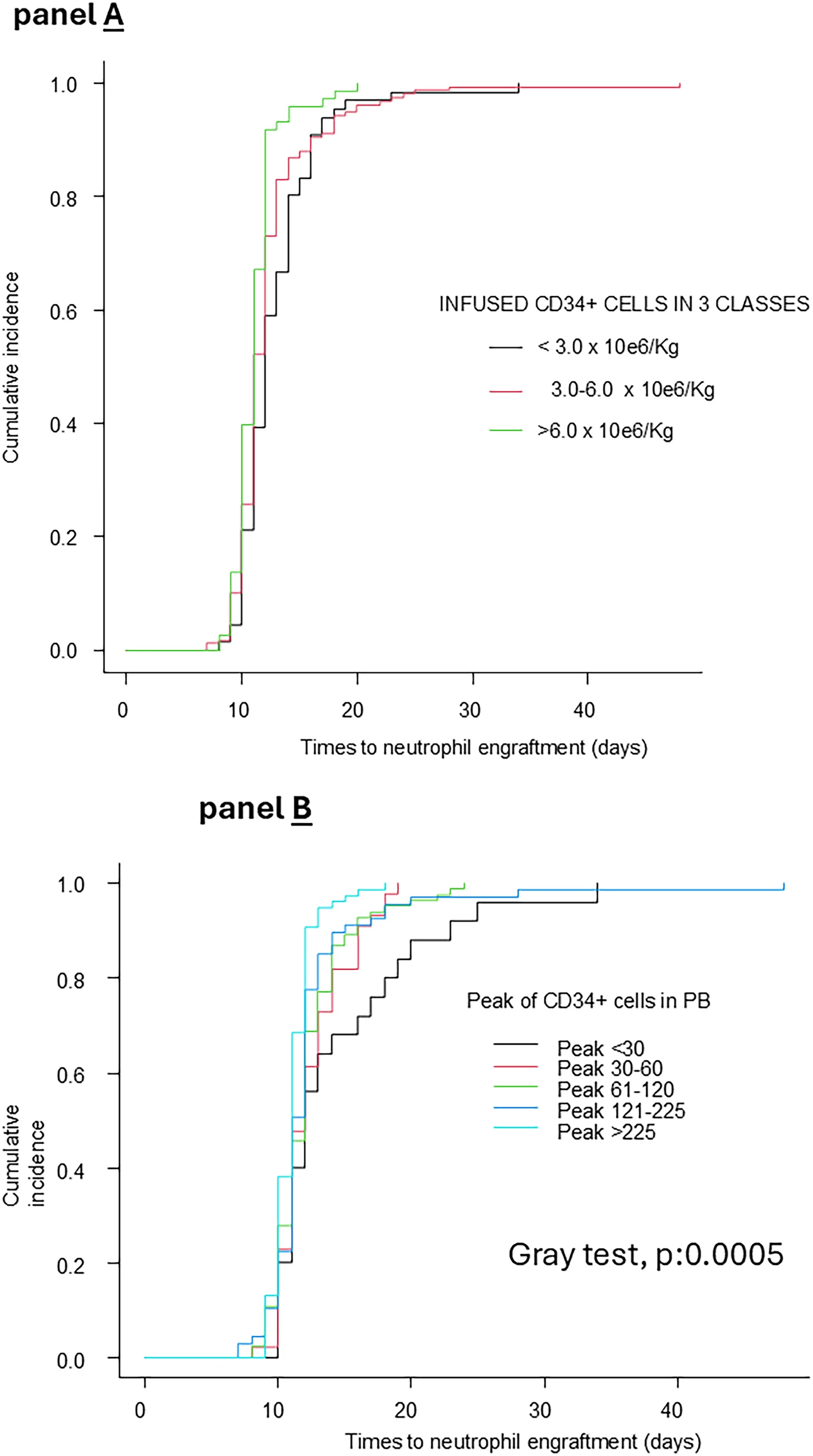
Cumulative incidence of engraftment in groups identified by infused CD34+ and by the peak of CD34+ in PB. Panel **(A)**: Cumulative engraftment incidence was evaluated in the three groups defined by infused CD34+/Kg. Differences were statistically significant (Gray test, p: 0.001). Panel **(B)**: Cumulative engraftment incidence was evaluated in the five groups defined by the peak of CD34+/µl in PB. Differences were statistically significant (Gray test, p: 0.0005).

In the Cox proportional hazard analysis for times to event, CD34+ cell dose resulted in an important effect when assessed as a continuous datum (HR: 1.048; 95% CI, 1.014-1.084), (p=0.005). When amount of CD34+ cells infused was analysed as a categorical variable (3 groups), with respect of the reference group (CD34+ > 6 x 10^6/kg), the first group (CD34+ < 3 x 10^6/kg) had a HR of 0.633 (95% CI, 0.454-0.883), p= 0.007; the group 2 (CD34 + 3–6 x 10^6/kg) had a HR of 0.729 (95% CI, 0.552-0.963), p= 0.025; therefore the event (engraftment) was registered in a significantly longer time in both groups compared with the reference group.

### Relevance of the peak of CD34+ cells in PB during mobilisation for neutrophil engraftment

The same set of patients was studied for neutrophil engraftment in groups defined by the CD34+ peak in PB reached before apheresis. The median CD34 peak was 120 cells/µL; percentile distribution was as follows: upper 75% threshold was 225 cells/µL; lower 25% threshold was 60 cells/µL; lower 10% threshold was 30 cells/µL. Accordingly, five groups were defined: group A (<30 cells/µL, n = 28), group B (30–60 cells/µL, n = 45), group C (61–120 cells/µL, n = 85), group D (121–225 cells/µL, n = 68), and group E (>225 cells/µL, n = 76).

Neutrophil engraftment in group A was 14.6 days (range 10-34); in group B was 12.4 days (8-19); in group C was 12.2 days (range 8-24); in group D was 12.7 days (range 7-48) and in group E was 11.0 days (range 9-18), (Kruskal Wallis test, p= 0.001). **Figures 1B**, **2B**.

When analysed as a continuous variable in a Cox proportional hazards model, the PB CD34+ peak was significantly associated with engraftment (p = 0.0002). When the PB CD34+ peak was expressed as 100 cells/µL, the HR was 1.069 (95% CI, 1.034-1.106).

Stratification of peak CD34+ cell counts into five groups and analysis using Cox proportional hazards modelling showed significantly delayed engraftment in groups with lower peak CD34+ counts compared with the reference group (group E). Using group E (PB CD34+ >225 cells/µL) as reference, hazard ratios were: group A, 0.428 (95% CI, 0.268–0.683; p = 0.0004); group B, 0.605 (95% CI, 0.416–0.879; p = 0.008); group C, 0.643 (95% CI, 0.469–0.881; p = 0.005); and group D, 0.621 (95% CI, 0.443–0.870; p = 0.005).

### Correlation of CD34+ cell peak and infused CD34+ with neutrophil engraftment

Both variables were correlated with times to neutrophil engraftment: infused CD34+ cells showed a coefficient of -0.117 (p=0.04); the CD34 peak showed a coefficient of -0.135 (p=0.01). After log transformation, the correlation coefficient for the PB CD34+ peak was -0.279 (95% CI, -0.381 to -0.169), whereas that for Infused CD34+ cells was -0.179 (95% CI, -0.287 to -0.065).

Infused CD34+ were correlated with PB CD34+ peaks (correlation coefficient +0.471; p= 0.0001).

### Identification of other factors important for engraftment

We studied whether other factors might have affected engraftment times and identified G-CSF use as a significant factor. In the univariate Cox proportional hazards model, not using G-CSF was associated with an HR of 0.665 (95% CI, 0.514-0.860; p = 0.001). The difference was significant when assessed using the Mann-Whitney test (p = 0.04).

Diagnosis was not significantly associated with neutrophil engraftment; indeed, the mean neutrophil engraftment was 12.3 days in the Multiple Myeloma group, compared with 12.5 days in the HD group and 12.0 days in the NHL group. In the univariate Cox proportional hazards model, the NHL group had an HR of 1.118 (p = 0.45) compared with the reference group (MM), and HD had an HR of 1.133 (p = 0.52).

Gender was not associated with neutrophil engraftment; the neutrophil engraftment was 12.0 in females and 12.3 in males. In the univariate Cox proportional hazards model, the female group had an HR of 1.115 (95% CI, 0.885-1.405) (p = 0.35) relative to the reference group (males), Mann-Whitney test, p = 0.13.

The 4 different mobilisation types (G alone; CTX plus G; DHAP plus G; other chemo) were evaluated for differences in neutrophil engraftment time using Kruskal-Wallis test, and resulted not important (p=0.15).

Number of apheresis performed: 252 patients received 1 apheresis and 50 patients received two or 3 apheresis. A higher number of apheresis performed was significantly associated to longer neutrophil engraftment: 12.0 days in patients receiving one apheresis versus 13.2 days in patients receiving two apheresis (Mann-Whitney test 0.03). Patients receiving two apheresis, however, had lower infused CD34 dose (3.8 x10^6/kg versus 4.1 x10^6/kg) and lower PB CD34 peak (41.1 cells/µL versus 133.2 cells/µL) in respect to patients receiving one apheresis.

Finally, patient age at transplantation was not significantly associated with engraftment time. In patients below 60 years, the mean engraftment time was 12.4 days (7-48), while in patients over 60 years, it was 11.9 days (8-25). This difference did not reach statistical significance (Mann-Whitney, p=0.98). Consistently, age was not associated with engraftment time when analysed as a continuous variable in a Cox proportional hazards model (HR 0.996; 95% CI, 0.986-1.007; p= 0.49).

We compared the five Groups identified by peak CD34 in PB (group A, B, C, D, E) with respect to age, distribution of diagnoses, and dose of infused CD34+ cells. Mean age did not differ across Groups (Kruskal-Wallis test, p = 0.44). The distribution of diagnoses was also comparable among groups (chi-square test, p = 0.18). However, as a consequence of the correlation among CD34+ peak in PB and infused CD34+ cells, in groups identified by PB peak of CD34+ cells, the infused CD34+ cell dose differed across groups: Group A were infused with a mean of 3.5 x 10^6/kg (2.1-6.0); Group B with a mean of 3.5 x 10^6/kg (1.4-9.0); Group C with a mean of 4.1 x 10^6/kg (2.0-10.6); Group D with a mean of 5.5 x 10^6/kg (2.0-13.2); Group E with a mean of 7.5 x 10^6/kg (2.7-26.5), (Kruskal-Wallis test p= 0.0001). Next, we assessed the independent contribution of the PB CD34+ peak using a multivariable analysis.

### Multivariable analysis

The use of G-CSF, the number of apheresis performed, the PB CD34+ peak (continuous data), and the infused CD34+ (continuous data) were evaluated in the multivariable analysis; PB CD34+ peak (HR 1.051; 95% CI, 1.009-1.095; p= 0.01) together with the use of G-CSF (HR 0.702; 95% CI, 0.539-0.914; p= 0.008) remained significantly associated with engraftment whereas infused CD34+ cell dose and number of apheresis were not.

Infused CD34+ cell dose across 3 categories and PB CD34 peak across 5 categories were also evaluated in the multivariable model, together with post-transplant G-CSF use. PB CD34+ peak across 5 categories remained significantly associated with engraftment, whereas infused CD34+ cell dose across 3 categories was not. Post-transplant G-CSF use also remained significantly associated with time to neutrophil engraftment (**Table 2****).** Although the two predictors, PB CD34+ peak and the infused CD34+ cells, were correlated (r = 0.471) the collinearity did not inflate the variance of the estimates: the maximum Variance Inflation Factor (VIF) was 1.40.

**Table 2:** Multivariate analysis.

Variable	Class	Hazard ratio	95% CI	p
infused CD34 <3 x10^6/kg	(class 1)	0.770	0.517-1.148	0.20
infused CD34 3–6 x 10^6/kg	(class 2)	0.928	0.684-1.259	0.63
infused CD34 > 6 x10^6/kg	(class 3)	Reference group		
PB CD34 peak below 30 cells/µL	Group a	0.489	0.292-0.817	0.006
PB CD34 peak 30–60 cells/µL	Group b	0.774	0.508-1.179	0.23
PB CD34 peak 61–120 cells/µL	Group c	0.690	0.489-0.974	0.03
PB CD34 peak 121–225 cells/µL	Group d	0.674	0.478-0.949	0.02
PB CD34 peak > 225 cells/µL	Group e	Reference group		
USE of G-CSF	no CSF	0.685	0.525-0.894	0.005
USE of G-CSF	yes CSF	Reference group		

A low CD34 peak in PB was a marker for PLX use. To rule out that PLX per se was the reason for delayed engraftment in patients with a low CD34+ peak, we repeated the analysis excluding patients treated with PLX. Among patients not treated with PLX, the PB CD34+ peak (as a continuous variable) remained significant in the multivariate analysis, along with G-CSF use, as shown in **Table 3**.

**Table 3:** Multivariable Cox proportional hazard analysis for neutrophil engraftment in the stratum “no PLX”.

Variable		Hazard ratio	95% CI	p
PB CD34+ peak		1.110	1.021-1.207	0.01
Infused CD34+		1.022	0.981-1.064	0.29
Use of G-CSF (no G-CSF)		0.750	0.573-0.982	0.03

### Interaction between PB CD34+ peak and post-transplant G-CSF

Since our multivariate analysis indicates that the CD34+ peak and G-CSF are the two relevant factors in determining differences in neutrophil engraftment, we then explored whether the degree of association between PB CD34+ peak and neutrophil engraftment varies with post-transplant G-CSF administration. In a linear model using log10-transformed PB CD34+ peak, post-transplant G-CSF, and their interaction, the interaction was statistically significant (β = 2.61, p = 0.002), indicating that the relationship between PB CD34+ peak and engraftment time was modified by post-transplant G-CSF use.

In patients who did not receive post-transplant G-CSF, higher CD34+ peak values were associated with shorter engraftment times, with a slope (β = -3.75; 95% CI = -5.65 to -1.85; R² = 0.14; Spearman ρ = -0.32, p = 0.002). In patients who received post-transplant G-CSF, the same inverse association was still present but was weaker (β = -1.14; 95% CI = -1.79 to -0.49; R² = 0.06; Spearman ρ = -0.20, p = 0.006) (**Figure 3**).

**Figure 3:**
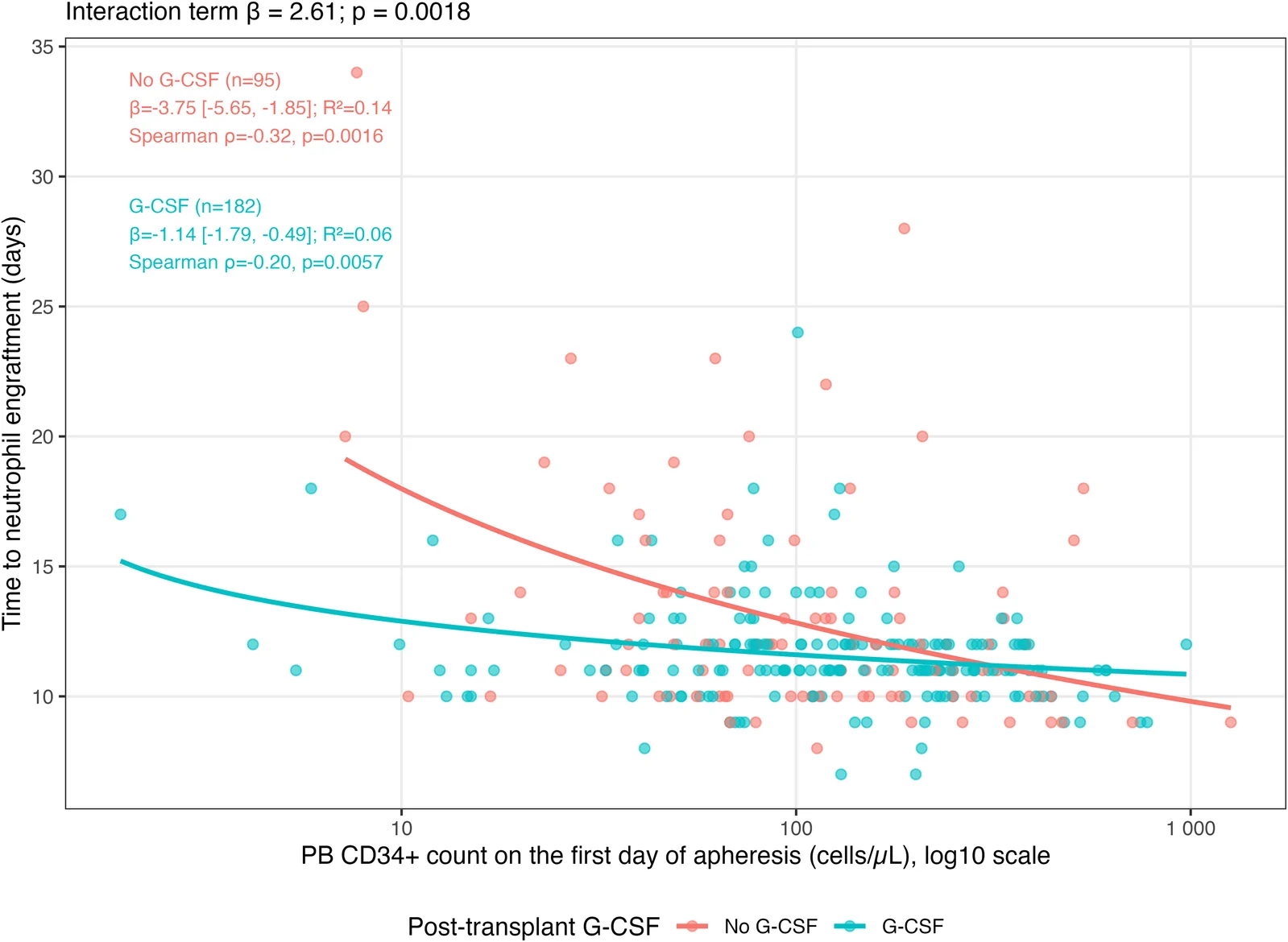
Association between PB CD34+ peak and neutrophil engraftment time according to post-transplant G-CSF use. A significant interaction depending on the use of G-CSF is present. PB CD34+ peak is shown on a log10 scale.

Overall, these findings suggest that post-transplant G-CSF partially reduces the impact of mobilisation quality, as reflected by CD34+ peak, on subsequent neutrophil recovery.

### Clinical usefulness of CD34+ peak in predicting delayed neutrophil engraftment

To study the clinical usefulness of the PB CD34+ peak reached during mobilisation, we evaluated its predictive value for neutrophil engraftment delayed beyond day 16.

A delay in neutrophil engraftment was observed in 28.5% (8/28) of patients who had a PB CD34+ cell peak below 30 cells/µL, in 17.7% (8/45) of patients having a peak 30–60 cells/µL; in 10.5% (9/85) of patients having a peak 61–120 cells/µL; in 10.2% (7/68) of patients with a peak 121–225 cells/µL and only in 2.6% (2/76) of patients having a peak over 225 cells/µL (Chi square test, p= 0.002).

When this outcome was studied in relation to infused CD34+, the following results were obtained:

Delayed neutrophil engraftment occurred in 16.1% (11/68) of patients receiving <3 × 10^6^ CD34+ cells/kg, 11.8% (19/160) of those receiving 3–6 × 10^6^ CD34+ cells/kg, and 5.4% (4/74) of those receiving >6 × 10^6^ CD34+ cells/kg (chi-square test, p = 0.11).

The value of PB CD34 peak as a predictor of delayed engraftment was also assessed using ROC analysis, yielding an AUC of 0.695 (95% CI, 0.599-0.791). In comparison, using the infused CD34+ cells/kg, the AUC had a significantly lower predictive value, with AUC 0.576 (95% CI, 0.48-0.673), p=0.01 (**Supplementary Figure 1**).

### Joint distribution of PB CD34+ peak and infused CD34+ cell dose

Using combined clinical information from the PB CD34+ peak and the infused CD34+ cell dose, we analysed engraftment based on the joint distribution of these two variables.

Median engraftment time was longest in patients with the lowest PB CD34+ peak values, especially when associated with a lower infused CD34+ dose (**Figure 4A**).

**Figure 4:**
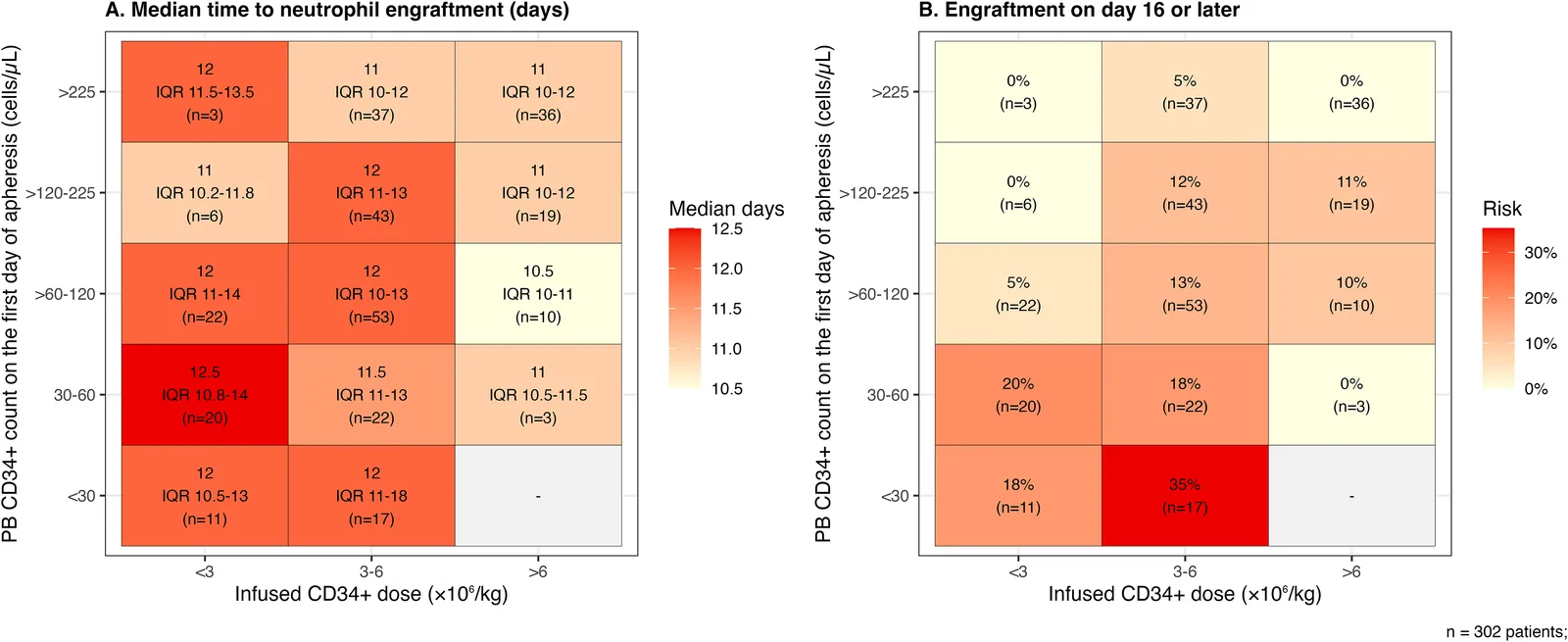
Heat maps: engraftment time according to the combined PB CD34+ peak class and infused CD34+ dose class. Panel **(A)**: median engraftment times. Panel **(B)**: Delayed engraftment beyond day 16. **(A)** Median neutrophil engraftment time according to combined PB CD34+ peak class and infused CD34+ dose class. Each cell reports median engraftment time, IQR, and sample size. Note as the subgroup having a lower PB peak of CD34+ (<30 CD34+ cells/µL) during mobilisation had the worst results in terms of neutrophil engraftment. **(B)** Risk of delayed neutrophil engraftment (>16 days) according to combined PB CD34+ peak class and infused CD34+ dose class. Each cell reports the percentage of delayed engraftment and sample size.

A similar pattern was observed for delayed engraftment (> 16 days). The highest risk was observed in patients with a PB CD34+ peak <30 cells/µL, with delayed engraftment rates of 35% in those infused with < 3 × 10^6 CD34+ cells/kg and 23% in those infused with 3-6 × 10^6 CD34+ cells/kg (**Figure 4B**).

Within the homogeneous stratum of patients infused with CD34 + 3–6 x 10^6/kg, the subgroups identified by different PB CD34+ peak had a neutrophil engraftment times of statistical significance (p=0.078) (**Figure 5**).

**Figure 5:**
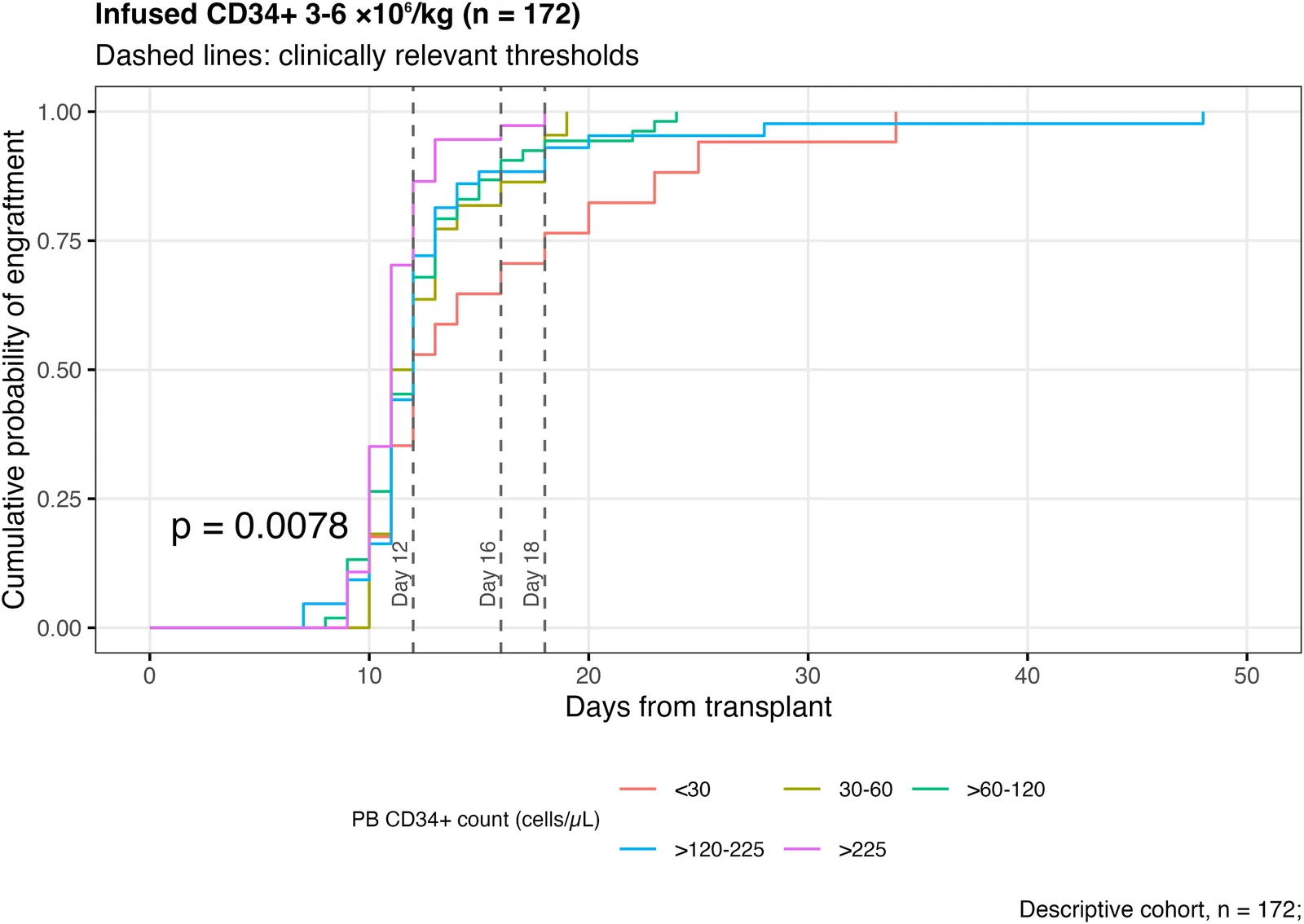
Curves are shown separately for each infused CD34+ dose category. P values refer to comparisons across PB CD34+ peak classes within each infused dose stratum.

An analysis using the threshold of 60 cells/u/L was also done, and the group with a PB CD34+ peak <60 cells/µL had a higher risk of delayed engraftment than patients with a PB CD34+ peak >60 cells/µL, 21.9% versus 7.8% (p=0.0009), (**Supplementary Table 1**). Furthermore, the risk of delayed engraftment was higher in those with low PB CD34 peak (< 60 cells/µL) irrespective of dose of infused CD34+ cells: delayed engraftment was observed in 30% (6/20) of patients who were infused with > 4x10^6 CD34+ cells/kg and who had a PB CD34+ cells peak < 60 cells/µL and in 7.6% (11/144) of patients infused with > 4 x10^6 CD34+ cells/kg and who had a PB CD34+ peak > 60 cells/µL (Chi-square: P = 0.002), (**Supplementary Table 1**). In **Figure 6** the median engraftment time and the risk of delayed engraftment according to PB CD34+ cell peak and to use of G-CSF is illustrated.

**Figure 6:**
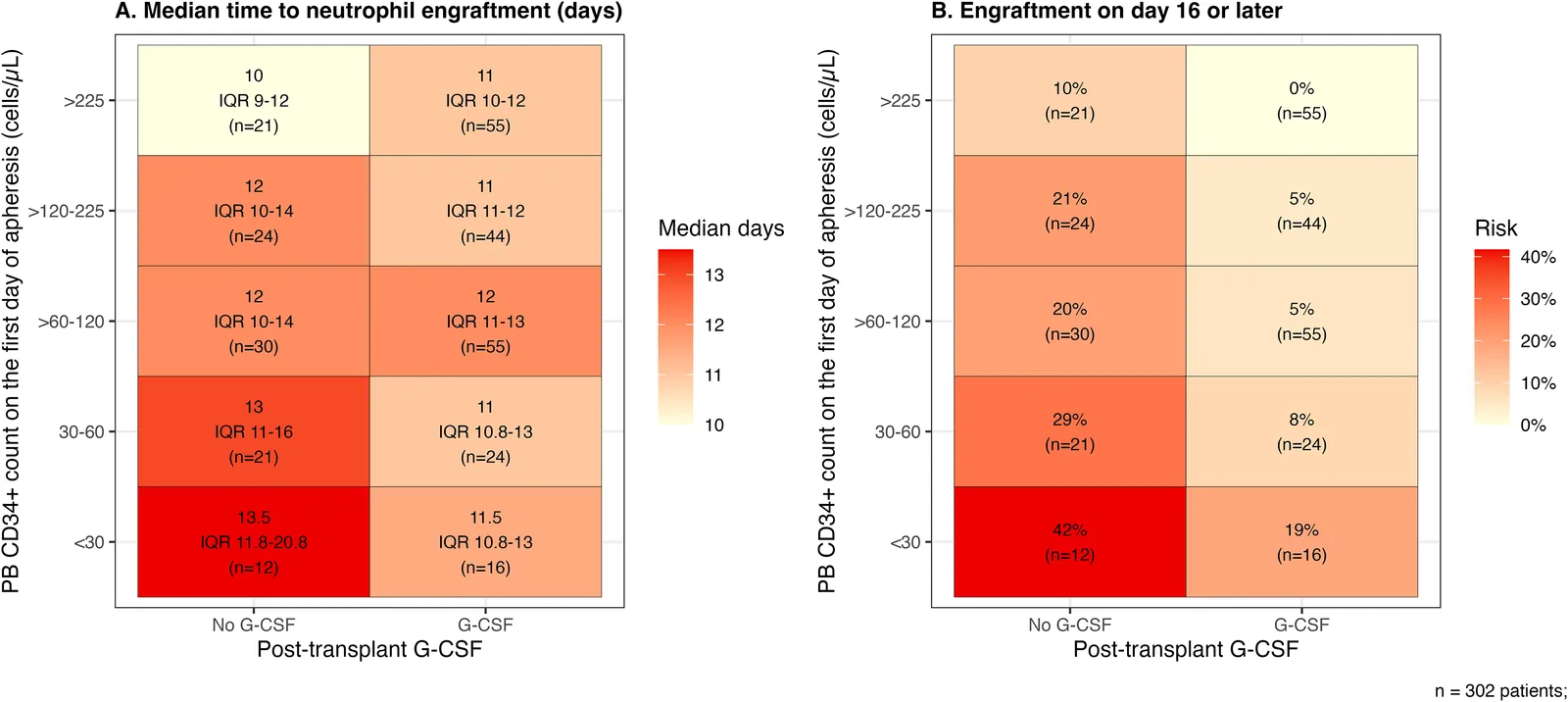
Heat maps: engraftment times according to PB CD34 peak and use of G-CSF. Panel **(A)**: median engraftment times. Panel **(B)**: Delayed engraftment beyond day 16th.

## Discussion

An adequate HSC inoculum is required to safely deliver high-dose therapy followed by autologous transplantation in patients with selected hematologic malignancies. It is generally accepted that within this scope, the minimum dose of infused CD34-positive cells should be ≥ 2 × 10^6/kg (2–5). Delayed engraftment has been reported with CD34+ cell doses below 2 × 10^6/kg (7, 15).

Given this minimum threshold, a successful apheresis harvest of PBSC cells is defined as a CD34+ content > 2 × 10^6/kg. This can be achieved in 90-95% of patients, depending on diagnosis, age, and previous treatments (16), but cases that do not reach this CD34+ cell quota are considered failures.

Despite the rate of non-mobilisation decreasing after the introduction of agents that interfere with the CXCR4/SDF1 axis (16, 17), around 5-10% of patients, depending on the underlying diagnosis, still do not achieve successful mobilisation and therefore cannot receive HD chemotherapy (18, 19).

The threshold dose of CD34+ cells (2 x 10^6/kg) has usually been determined by correlating time to engraftment with the dose of infused CD34+ per kg of actual body weight. Recently, an improved correlation between CD34+ dose and neutrophil engraftment has been observed when recipient blood volume, rather than recipient body weight, was used (20).

In addition to the total CD34+ cell dose, CD34+ cell subsets have also been investigated. A CD34+ Thy1+ subset is correlated with long-term engraftment (12). The CD34+/CD38- subset of HSC has been reported to be important for rapid neutrophil engraftment (9, 21), although conflicting results have been published on this issue. In fact, the total CD34+ dose has been reported to better predict outcomes than any CD34+ subset (22).

In the mouse, purified early stem cells have been shown to confer rapid engraftment (23).

However, in addition to HSC quantity, other factors related to the so-called “HSC quality” should be considered when interpreting engraftment. Cryopreservation, in itself, alters many metabolic aspects of HSC (24), and the viable number of CD34+ cells after cryopreservation is more useful than the total number of CD34+ cells counted before cryopreservation (25).

The marrow microenvironment also plays a role in engraftment, and damage to the microenvironment has been proposed to explain delayed platelet engraftment after autologous HSC transplant in patients who received prior cyclophosphamide (26).

We have found that patients transplanted with the same number of CD34 cells may have different engraftment times depending on the peak CD34 level in PB during mobilisation. These findings are consistent with previous reports, indicating that better engraftment is achieved in patients who reach the CD34+ quota in only 1 or 2 apheresis procedures, a surrogate marker of high-grade CD34 mobilisation (12). From a conceptual point of view, our data strongly suggest that, at least in patients with Multiple Myeloma or Lymphoma, the degree of mobilisation is linked to the quality of the mobilised HSC.

In our centre, collection was guided by an algorithm based on the CD34+ cell count in PB (13). Under such a target-driven strategy, the collected CD34+ dose is determined not only by individual mobilisation biology but also by adjustments made during the process, including the timing of collection relative to PB CD34+ kinetics, the number of procedures performed, and the use of rescue mobilisation with plerixafor. These adjustments will reduce inter-individual variability in the amount of CD34+ collected. They may, however, introduce saturation effects around the target yield, thereby limiting the ability of the collected CD34+ dose to discriminate relevant aspects in the biology of the mobilisation process. In contrast, the PB CD34+ peak is measured before collection and directly reflects the integrated output of marrow reserve/responsiveness and niche-dependent retention/egress dynamics; accordingly, it may retain a stronger association with the quality of HSC inoculum, thus allowing for a better prediction of time to neutrophil engraftment even after accounting for diagnosis and post-transplant G-CSF use.

However, the retention of PB CD34^+^ peak, but not infused CD34^+^ dose, in the multivariable model should not be interpreted as evidence of a greater causal importance of PB CD34^+^ cells. It should be considered in this respect that cases were selected in such a way to have a quite small range in the infused CD34+ cells while PB CD34 peak was allowed to have a very wide distribution among cases, and this increased its statistical power.

Furthermore, the PB CD34^+^ peak, when considered in isolation, is not suitable for judging whether the total number of harvested CD34+ cells is adequate. In fact, the peak CD34+ cell count in PB does not reflect the absolute CD34+ content of the apheresis harvest. However, the peak CD34+ cell count in PB carries additional information and may be useful for assessing the suitability of the inoculum, in addition to the total CD34+ cell count. In this context, joint stratification by PB CD34+ peak and infused CD34+ dose showed that PB CD34+ peak adds clinically relevant information within the same infused-dose categories. In particular, very low PB CD34+ peak values identified patients with slower engraftment and a higher risk of delayed engraftment, even when the infused CD34+ dose was at a typically used level. It is noteworthy that patients with a low CD34+ peak (< 60) and an apparently sufficient infused dose of CD34+ cells have a risk of prolonged neutropenia of up to 21.9%. The limit of our study is that the selection of the population, only adult patients and only patients receiving inoculum harvested in a single mobilisation course, reduces the general applicability of our findings. Thus, these data require external validation and further studies to determine whether PB CD34+ peak is clinically relevant relative to other clinical aspects of HSC transplantation and for predicting platelet engraftment.

In conclusion, from a practical perspective, the degree of mobilisation may be an additional parameter to consider alongside the total harvested CD34+ cell dose when assessing the adequacy of the CD34+ inoculum before HPC transplantation.

## Data Availability

The raw data supporting the conclusions of this article will be made available by the authors, without undue reservation.
