# Supplementary material for: Peripheral blood CD34+ peak during mobilisation as an additional parameter to assess PBSC inoculum adequacy autologous HSCT
Source: Front Immunol. 2026 Oct 16;17:1935217. doi: 10.3389/fimmu.2026.1935217 (PMC13719430; doi:10.3389/fimmu.2026.1935217)
Supplement: Supplementary file 1 [file Table1.doc]

Supplementary Table 1

Delayed engraftment according to PB CD34+ cell peak (< 60/µl or > 60/µl) and according to infused dose of CD34+ (< 4 x 10^6/kg or > 4 x 10^6/kg)

|  | A | B |  | C | D |
| --- | --- | --- | --- | --- | --- |
|  | PB peak ≤ 60 /µl and infused  CD34+ < 4.0 x 10^6 /kg | PB peak ≤ 60 /µl and Infused CD34+ > 4.0 x 10^6/kg |  | PB peak > 60/µl  and Infused  CD34+ <4.0 x 10^6/kg | PB peak > 60/µl  and Infused  CD34+ >4.0 x 10^6/kg |
| N | 53 | 20 |  | 85 | 144 |
| Delayed Neutrophil Engraftment | 21.9% | | A+B vs C+D  P=0.0009 | 7.8% | |
|  | 18.8% (10/53) | 30% (6/20) | A+B vs C+D  P=0.0009 | 8.2% (7/85) | 7.6% (11/144) |
| Use of PLX | 38.8% | 31% | A+B vs C+D  P=0.003 | 1.1% | 1.4% |
| Infused CD34+ cells (mean) | 2.8 (1.4-4.0) | 5.3 (4.1-9.0) | A+B vs C+D  P=0.0001 | 3.1 (2.0-4.0) | 7.1 (4.0-26.5) |
| PB Peak CD34  Cells/µL  (range) | 32.7  (1.9-59.0) | 34.6  (5.4-56.6) | A+B vs C+D  P=0.0001 | 124.5  (60.0-409.0) | 279.4  (64.0-3471.0) |
| Apheresis  Session  (Mean) | 1.5  (1-2) | 1.4  (1-3) | A+B vs C+D  P=0.0001 | 1.1  (1-2) | 1.0  (1-2) |
| Neutrophil engraftment  Days (range) | 12.7  (8-34) | 14.1  (10-25) | A+B vs C+D  P=0.008 | 12.1  (8-24) | 11.9  (7-48) |
